# Oxygenation Profiles of Human Blood, Cell Culture Medium, and Water for Perfusion of 3D-Bioprinted Tissues using the FABRICA Bioreactor Platform

**DOI:** 10.1038/s41598-020-64256-1

**Published:** 2020-04-29

**Authors:** Angela M. Chen, Matthew Lashmet, Abdulkadir Isidan, Jane L. Sterner, Julia Walsh, Cutter Koehler, Ping Li, Burcin Ekser, Lester Smith

**Affiliations:** 1 Division of Transplant Surgery, Department of Surgery, Indiana University School of Medicine, Indianapolis, IN USA; 2 Department of Radiology and Imaging Sciences, Indiana University of School of Medicine, Indianapolis, IN USA; 3 3D Bioprinting Core, Indiana University School of Medicine, Indianapolis, IN USA

**Keywords:** Regenerative medicine, Tissue engineering, Engineering

## Abstract

Persistent and saturated oxygen distribution from perfusion media (i.e., blood, or cell culture media) to cells within cell-dense, metabolically-active biofabricated tissues is required to keep them viable. Improper or poor oxygen supply to cells within the tissue bulk severely limits the tissue culturing potential of many bioreactors. We added an oxygenator module to our modular FABRICA bioreactor in order to provide stable oxygenation to biofabricated tissues during culture. In this proof of concept study of an oxygenated and perfused bioreactor, we characterized the oxygenation of water, cell culture medium, and human blood in the FABRICA as functions of augmenting vacuum (air inlet) pressure, perfusion (volumetric flow) rate, and tubing/oxygenator components. The mean oxygen levels for water and cell culture media were 27.7 ± 2.1% and 27.6 ± 4.1%, respectively. The mean oxygen level for human blood was 197.0 ± 90.0 mmHg, with near-physiologic levels achieved with low-permeability PharMed tubing alone (128.0 ± 14.0 mmHg). Hematologic values pre- and post-oxygenation, respectively were (median ± IQR): Red blood cell: 6.0 ± 0.5 (10^6^/μL) and 6.5 ± 0.4 (10^6^/μL); Hemoglobin: 17.5 ± 1.2 g/dL and 19.2 ± 3.0 g/dL; and Hematocrit: 56.7 ± 2.4% and 61.4 ± 7.5%. The relative stability of the hematologic parameters indicates that blood function and thus blood cell integrity were maintained throughout oxygenation. Already a versatile research tool, the now oxygenated FABRICA provides easy-to-implement, *in vivo*-like perfusion and stable oxygenation culture conditions *in* vitro semi-independently of one another, which means the bioreactor has the potential to serve as a platform for investigating the behavior of 3D tissue models (regardless of biofabrication method), performing drug toxicity-testing, and testing pharmaceutical efficacy/safety.

## Introduction

Recent 3D bioprinting/biofabrication advances allow generation of scaffold-free engineered tissue constructs with customizable 3D design and high cell density^1–4^. These engineered tissues are comprised of cells in contact with one another and with extracellular matrix (ECM) in dense 3D structures, thus making them similar to natural tissues *in vivo* with the same requirements of controlled, well-distributed oxygenation, nutrition, mechanical (shear, compressive, and tensile) forces, temperature, pH, etc.^5–15^ so that all cells receive proper metabolic and stimulatory support. Compared to cells cultured in 2 dimensions, engineered tissues with 3D structures, biofabricated by means of bioprinting or other methods, can plausibly be tuned to meet specific genetic, cellular, structural, extracellular, and metabolic parameters, and thus have incredible potential to provide tissues for clinical implants, research models, and drug testing platforms. Importantly, given that media (cell culture media or blood), used to perfuse biofabricated tissue cultured in bioreactors, is one constituent providing multiple critical metabolic factors (convective heat, nutrients, shear stress, and oxygen), the conditions of perfused media should be carefully controlled. Most critically, persistent and saturated distribution of oxygenated perfusion media (i.e., blood, or cell culture media) to cells within cell-dense, metabolically-active tissues, biofabricated or otherwise, is required for cellular respiration to occur and thus to keep cells viable^8,10,12,13,15–18^. In mammals, this is achieved by air mixing with the blood in the lungs and then being distributed to tissues by means of the vasculature (vascular perfusion). However, improper or poor provision of oxygen to cells within the tissue bulk severely limits the ability of many bioreactors to support tissue culture^5–8,17,19,20^ so that all cells receive proper metabolic and stimulatory support. Compared to cells cultured in 2 dimensions, engineered tissues with 3D structures, biofabricated by means of bioprinting or other methods, can plausibly be tuned to meet specific genetic, cellular, structural, extracellular, and metabolic parameters, and thus have incredible potential to provide tissues for clinical implants, research models, and drug testing platforms. Importantly, given that media (cell culture media or blood), used to perfuse biofabricated tissue cultured in bioreactors, is one constituent providing multiple critical metabolic factors (convective heat, nutrients, shear stress, and oxygen), the conditions of perfused media should be carefully controlled. Most critically, persistent and saturated distribution of oxygenated perfusion media (i.e., blood, or cell culture media) to cells within cell-dense, metabolically-active tissues, biofabricated or otherwise, is required for cellular respiration to occur and thus to keep cells viable. In mammals, this is achieved by air mixing with the blood in the lungs and then being distributed to tissues by means of the vasculature (vascular perfusion). However, improper or poor provision of oxygen to cells within the tissue bulk severely limits the ability of many bioreactors to support tissue culture^8,10,12,13,15–18^. In mammals, this is achieved by air mixing with the blood in the lungs and then being distributed to tissues by means of the vasculature (vascular perfusion). However, improper or poor provision of oxygen to cells within the tissue bulk severely limits the tissue formation potentialability of many bioreactors to support tissue culture^5,6,8,9,17,19,20^.

While there have been successful attempts at building oxygenating bioreactors, there remain limitations. Many have demonstrated perfusion with just a single medium, be it cell culture medium or blood^5,21^. In addition, bioreactors in the field of biofabrication are often purpose built, meaning they are limited to accepting and culturing tissues of a single type (e.g., bone or liver, etc.)^22,23^, made by a particular biofabrication method (e.g., scaffold-free, rotating wall vessel, etc.)^5,11,24^, or of a particular structural design (tubular, disk-shaped, etc.)^5,25,26^. Many bioreactor platforms lack modularity which is needed to account for design updates or new modules intended to increase functionality. In our previous work, we demonstrated our ability to control and characterize perfusive flow applied to tissues of different types and designs cultured within our FABRICA bioreactor platform, a bioprinting method-independent tissue perfusion system^1,27^. However, the oxygenation status of the tissues cultured within the FABRICA was not characterized. In our effort to provide low-cost, simply-implemented, and well-characterized biofabrication methods, we decided in the current study to investigate FABRICA oxygenation and found that the materials and methods required to provide simple near-normoxic perfusion media depends on the type of media being perfused and the type of tubing being used. Herein, we describe simple methods for oxygenating cell culture media (at normoxic levels, defined here as the oxygenation level of blood when it leaves the lungs) delivered to the FABRICA basin and the simple design changes necessary to normoxically-oxygenate blood delivered to the same platform. Since the FABRICA is both bioprinting method and tissue design agnostic, and studying tissue oxygenation (i.e., oxygen distribution and tissue survival) is futile without first providing and characterizing media oxygenation, we decided to investigate oxygenation of the perfused media without including biofabricated tissues. The complexities associated with designing, fabricating, culturing, and analyzing complex tissues with perfusible structures (i.e., vascular-like channels) are vast enough that they deserve their own investigation in parallel with oxygenation, which we have begun^27^.

Here, we describe the development and characterization of a FABRICA bioreactor equipped with an oxygenator module and or tubing to provide flow rate-controlled, oxygen-enriched water, cell culture media, or human blood to biofabricated tissues. Combined with scaffold-free biofabrication methods and controlled nutrient flow through tissues within the bioreactor, the inclusion of stable oxygenation brings the FABRICA’s artificial *in vitro* microenvironment in closer fidelity to *in vivo* conditions, thus making it a useful tissue engineering platform for research and development. Moreover, the final implementation of the stably-oxygenated FABRICA system is simplified and generalizable for use with different tissue types and fabrication methods, making it a novel system for culturing engineered tissues *in vitro* with an *in vivo*-like environment.

## Methods

### Bioreactor Preparation

**Figure 1:**
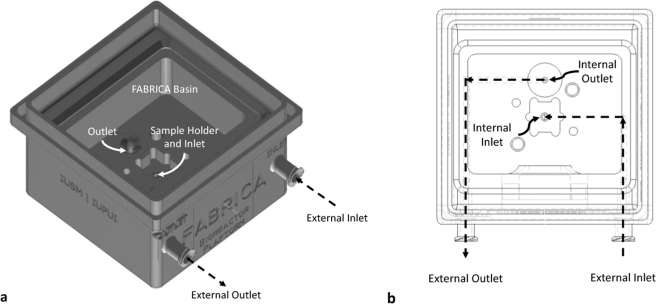
FABRICA bioreactor with integrated flow channels and luer lock connector ports. Left: Oblique 3D render of FABRICA. Right: Line render of FABRICA from top view.

We used the previously reported FABRICA Bioreactor Platform^1^ (Fig. 1), with some modifications. Briefly, the FABRICA was 3D printed from Dental LT Clear or Denture Teeth A2 resin (Formlabs, Somerville, MA, USA) using a Form 2 desktop 3D printer (Formlabs, Somerville, MA, USA) and post-processed per manufacturer guidelines. The FABRICA was then soaked in 70–100% ethanol overnight followed by air-drying, soaked in Milli-Q water overnight, and air dried before use. The FABRICA has inlet and outlet channels integrated into the bioreactor. The channels open into the FABRICA basin, which, during tissue perfusion, is filled with medium. The sample holder/internal inlet port is situated in the center of the FABRICA basin such that oxygenated medium flows directly to the tissue construct. The internal outlet port is situated on the floor of the FABRICA basin. The channels are connected by the external inlet and outlet ports to tubing outside of the FABRICA by locks or slip tubing. The tubing is connected to a peristaltic roller pump (Ismatec Reglo ICC, Cole- Parmer, Wertheim, Germany).

### General Oxygenation Set Up

**Figure 2:**
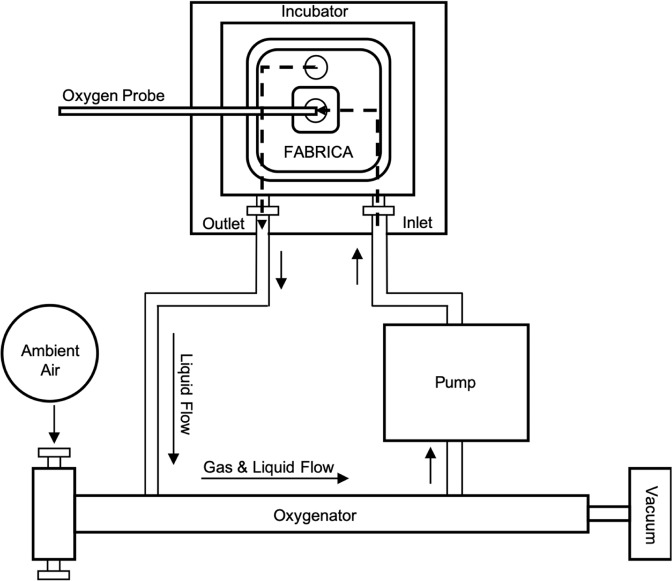
Schematic of FABRICA perfusion setup with FABRICA bioreactor, peristaltic pump, oxygenator, and NeoFox oxygenation sensor probe.

Silicone tubing (1.52 mm inner diameter, Cole-Parmer Ismatec, Wertheim, Germany) attached the oxygenator (Catamount Research and Development, Inc., St. Albans, Vermont, USA) to the external inlet and outlet ports of the FABRICA bioreactor^1^. Since silicone is oxygen-permeable, PharMed tubing (1.6 mm inner diameter, Cole-Parmer Ismatec, Wertheim, Germany) was used to test the low-permeability condition, with a short segment of silicone tubing used at the pump rollers to permit pumping. A peristaltic roller pump (Ismatec Reglo ICC, Cole-Parmer, Wertheim, Germany) was connected between the ports of the oxygenator and FABRICA, as shown in Fig. 2. When the oxygenator was implemented, additional tubing (3.2 mm inner diameter, Saint Gobain Tygon, Courbevoie, France) connected the house vacuum line to the gas outlet of the oxygenator through a pressure regulator. Pressure from the vacuum was used to draw ambient air into the oxygenator.

### Calibration for water and culture media oxygenation

Fluid oxygenation profile studies for both water and cell culture media (Dulbecco’s Modified Eagle Medium 1 g/L glucose [Gibco 11885-084, Grand Island, New York, USA], 10% FBS, and less than 1% supplement), were performed utilizing a NeoFox Phase Fluorometer (Ocean Optics, Inc., Largo, Florida, USA) fitted with an oxygen sensing probe via an optical cable. The oxygen sensor was inserted into the FABRICA through a custom lid and submerged into the internal inlet within the bioreactor. The NeoFox was connected to a Dell laptop (Dell, Inc., Round Rock, Texas, USA) through a USB cable and measurements were recorded using NeoFox-provided Viewer Software.

**Figure 3:**
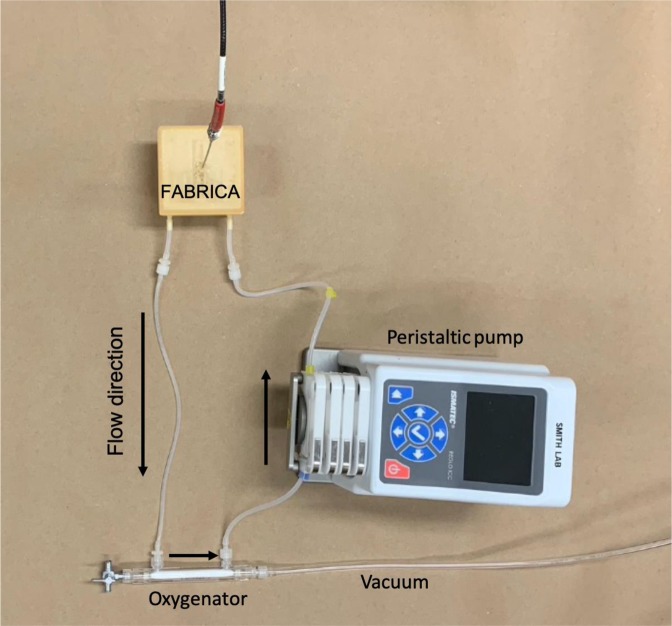
Photo of FABRICA perfusion setup with FABRICA bioreactor, peristaltic pump, oxygenator, and NeoFox oxygenation sensor probe connected by silicone tubing. For the oxygenator-free setup, the oxygenator and the vacuum are removed so that the outlet from the FABRICA goes directly into the pump.

The FABRICA bioreactor was placed in a cell culture incubator and acclimated to 37 °C and 8% carbon dioxide (CO_2_). Calibration measurements were taken at the FABRICA bioreactor internal inlet. To calibrate the NeoFox Oxygen Sensing Probe, oxygen-free water, oxygen-free culture media, equilibration water and equilibration culture media were prepared. The oxygen-free solutions were prepared using two grams of sodium sulfite in 50 mL of purified water or culture media. Equilibration solutions were produced by bubbling house air in 100 mL of water or culture media for half an hour or more. For each solution, a 33 mL volume was poured into the FABRICA bioreactor and the system was primed for ten minutes prior to calibration at 10 mL/min. The oxygen probe was inserted into the top of the removable lid of the FABRICA bioreactor such that the sensor was positioned at the internal inlet of the FABRICA (Figs. 2 and 3). Once the readings stabilized, an oxygen measurement was read and used as the zero percent level point for the calibration. The system was emptied and then flushed with 33 mL of Dulbecco’s phosphate-buffered saline (DPBS) for 10 minutes, ensuring that no remnants of the sodium sulfite remained. The system was then emptied again and filled with 33 mL of equilibration solution. Once the measurements had stabilized, an oxygen reading was read and used as the 20.9% oxygen calibration level for the oxygen-sensing probe.

### Oxygenated Water and Cell Culture Medium Perfusion Flow Profile Studies

Oxygenated perfusion/flow profile studies using the oxygenated FABRICA were performed using water or cell culture media pumped into the FABRICA bioreactor through its ports at 1, 3, 5, 7 and 10 mL/min at varying vacuum pressures (−3.39, −8.47, −16.93, −25.40, −33.86 kPa). At each flow rate and vacuum setting, the system was allowed to equilibrate for 10 minutes before a reading of SaO_2_ (oxygen saturation, %) was taken.

### Oxygenated Human Blood Perfusion Flow Profile Studies

A fluid oxygenation profile study was performed by flowing human leuko-reduced packed red blood cells through the FABRICA bioreactor. Blood for this research was obtained from the Indiana Blood Center or Indiana University Health Blood Bank. Blood types A−, A+, and O− were used. The oxygenation profile of human blood at several flow rates and vacuum pressures was determined using the FABRICA housed in an incubator at 37 °C and 8% CO_2_ and connected to the peristaltic pump outside of the incubator. Before perfusion, the blood was mixed with 1000 units of heparin per mL and stored on ice. Blood oxygen parameters were analyzed using the i-STAT Handheld Blood Analyzer (Abbott Laboratories, Inc., Chicago, Illinois, USA) and CG4+ or CG8+ Cartridges. Blood composition parameters, red blood cell count (RBC), hemoglobin (HGB), and hematocrit (HCT), were analyzed using an Element HT5 Hematology Analyzer (Heska, Loveland, Colorado, USA). Blood parameters recorded from the i-STAT were pH and partial pressure of oxygen in mmHg (PaO_2_).

The condition and composition parameters of the non-perfused blood were collected to establish a baseline status of the blood prior to perfusion. The non-perfused blood was incubated at 37 °C during the perfusion study and baseline values were obtained again at the conclusion of the experiment. For each set of flow rate trials, the blood analyzed at the previous pressure level was removed and a new volume of blood was injected into the FABRICA bioreactor and primed at 10 mL/min with less than 1 second of vacuum to help draw blood through oxygenator and then allowed to equilibrate for 10 minutes. Following priming during the silicone tubing with oxygenator condition, the blood was perfused through the system with −3.39, −8.47, −16.93, −25.40, and −33.86 kPa vacuum backpressure. For each vacuum pressure setting, the blood was perfused at 1, 3, 5, 7 and 10 mL/min for 10 minutes. Each flow condition was tested in triplicate. Following each set of flow rate trials, the blood condition was analyzed using the i-STAT and the hemoanalyzer.

### Statistical analysis

Statistical analysis was performed with Statistical Analysis Software University Edition 2018 (Cary, North Carolina, USA). The Kolmogorov-Smirnov test was used to test for normality. Data are presented as mean ± standard deviation (SD) for water and cell culture media and as median ± interquartile range (IQR) for blood and hematologic parameters. The Wilcoxon signed rank test was used to compare differences between pre- and post-oxygenation RBC, HGB, and HCT. Pearson’s correlation coefficient was used for water and media and Spearman’s correlation coefficient for blood to determine the relationship between flow rate and oxygenation across each of the three media when perfused. Two-way analyses of covariance with Tukey adjustment for multiple comparisons were used to determine the effect of flow rate and tubing condition on SaO_2_ for water and media and PaO_2_ for blood. Alpha level of 0.05 was used for statistical significance.

## Results

Overall oxygen levels for water and cell culture media were 27.7 ± 2.1% and 27.6 ± 4.1%, respectively. Water oxygen saturation for silicone tubing alone and silicone-with-oxygenator conditions were 29.1 ± 1.5% and 26.3 ± 1.7%, respectively. Cell culture media oxygen saturation for silicone tubing alone and silicone-with-oxygenator conditions were 24.1 ± 1.5% and 31.0 ± 2.8%, respectively.

The overall oxygen level for perfused blood was 197.0 ± 90.0 mmHg. Blood partial pressures of oxygen for PharMed tubing alone, silicone tubing alone, and silicone-with-oxygenator conditions were 128.0 ± 14.0 mmHg, 198.5 ± 11.0 mmHg, and 235.0 ± 19.0 mmHg, respectively. Oxygen levels for blood were interpreted as PaO_2_ instead of converting to SaO_2_. The oxygen dissociation curve follows a sigmoidal curve, and at PaO_2_ above 100 mmHg, SaO_2_ reaches the maximum of 100%^28^. Additionally, the i-STAT Blood Analyzer did not consistently provide a SaO_2_ value but did always provide an output for the PaO_2_.

**Figure 4:**
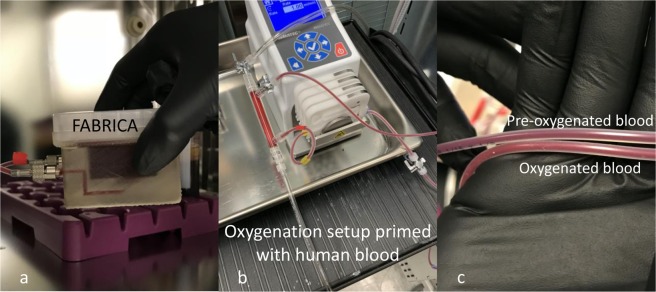
Blood oxygenation. (**a**) FABRICA bioreactor perfused with blood. (**b**) Oxygenation set up primed with blood. c. Inlet and outlet tubing to and from oxygenator, respectively, showing pre-oxygenated and oxygenated blood.

The oxygenation/perfusion setup pumped blood through the silicone tubing, oxygenator, pump, and FABRICA bioreactor with no issues and perfused blood exhibited a visible a color change due to oxygenation (Fig. 4).

**Figure 5:**
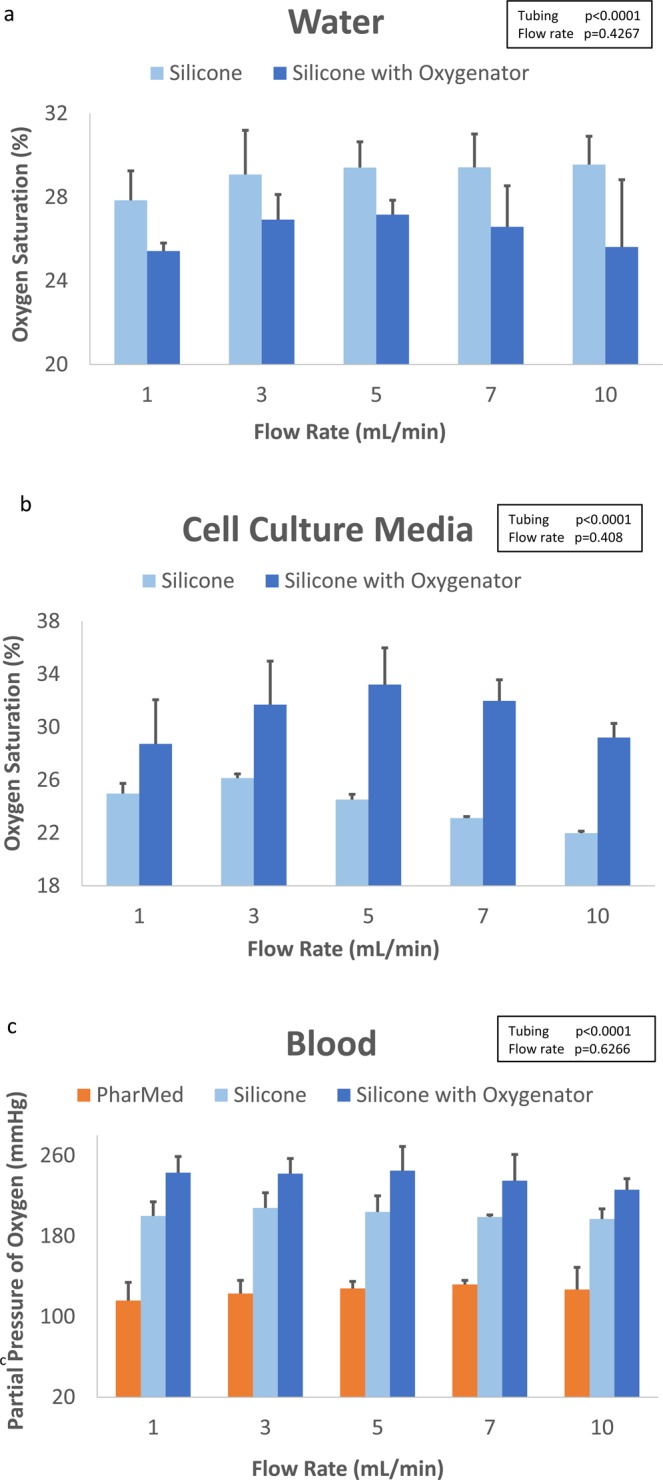
Summary graphs of perfusion medium oxygenation profiles per flow rate with various tubing and oxygenator conditions. For water and growth media, bars represent means and error bars represent standard deviation. For blood, bars represent medians and error bars represent interquartile range. (**a**) Oxygenation output for water. (**b**) Oxygenation output for growth medium. (**c**) Oxygenation output for blood.

Summary graphs for oxygenation in each medium at various volumetric flow rates and tubing and/or oxygenator conditions are shown in Fig. 5. All vacuum pressure conditions were tested, but for the purposes of the silicone with oxygenator condition, data at −25.40 kPa were chosen for analysis and comparison since this was the lowest vacuum condition that was able to sufficiently oxygenate without forming bubbles. In the non-perfused setting for blood (flow rate = 0 mL/min, vacuum pressure = 0 kPa), the median oxygen level was 64.0 ± 25.5 mmHg.

For water oxygenation profile studies, flow rate had a weak positive correlation with oxygenation (r = 0.1152, p = 0.5443). Alternatively, there were weak negative correlations between vacuum pressure and oxygenation for growth media (r = −0.1538, p = 0.4172) and blood (r = −0.1250, p = 0.3820) (not shown).

For water, there was a significant difference in oxygenation values between the silicone tubing alone and silicone-with-oxygenator conditions (p < 0.0001). However, oxygen differences between flow rates were non-significant (p = 0.4267). Cell culture media showed statistically significant differences between the means of oxygenation across tubing conditions (p < 0.0001) and flow rates (p = 0.0408), however individual comparisons between flow rates were non-significant. For blood, median oxygenation differences were significantly across the 3 tubing/oxygenator conditions (p < 0.0001) but non-significant across flow rates (p = 0.6266).

**Figure 6:**
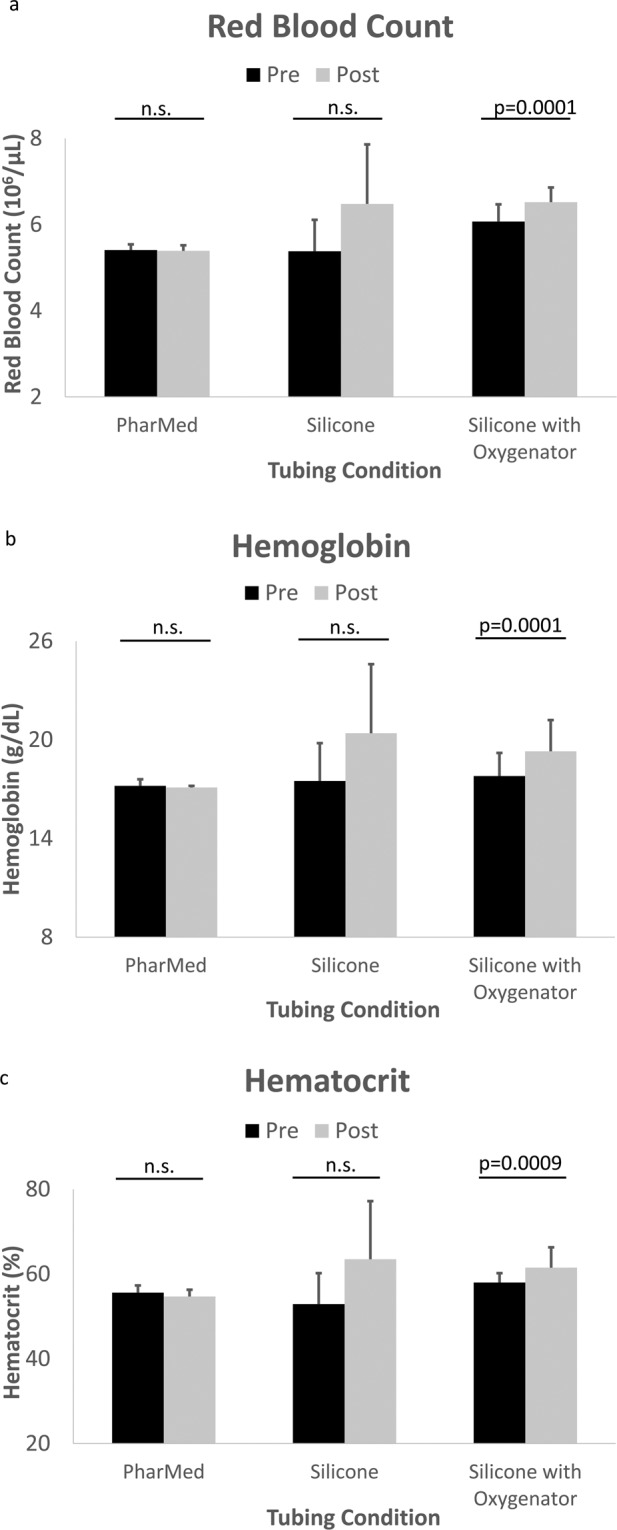
Hematologic parameters for blood across various tubing and/or oxygenator conditions, pre- and post-perfusion (PharMed n = 3 pairs, Silicone n = 3 pairs, Silicone with Oxygenator n = 15 pairs). Bars represent medians and error bars show interquartile range. (**a**) red blood cell count (10^6^/μL). (**b**) hemoglobin (g/dL). (**c**) hematocrit (%).

Hematologic values pre- and post-perfusion are shown in Fig. 6 (median ± IQR): 6.0 ± 0.5 (10^6^/μL) for RBC, 17.5 ± 1.2 g/dL for HGB, 56.7 ± 2.4% for HCT. Post-oxygenation hematologic values were (median ± IQR): 6.5 ± 0.4 (10^6^/μL) for RBC, 19.2 ± 3.0 g/dL for HGB, 61.4 ± 7.5% for HCT. For the silicone-with-oxygenator condition, all hematologic parameters were statistically significant after perfusion (p = 0.0001 for RBC, p = 0.0001 for HGB, p = 0.0009 for HCT). In the silicone tubing only and PharMed tubing only conditions, RBC, HGB, and HCT were not significantly changed after perfusion (p = 0.25 for RBC, p = 0.25 for HGB, p = 0.25 for HCT for silicone; p = 0.50 for RBC, p = 1.0 for HGB, p = 1.0 for HCT for PharMed).

## Discussion

### The FABRICA can be used to oxygenate water, cell culture media, and blood with ambient air

The water, cell culture media, and blood were oxygenated using a FABRICA bioreactor, with ambient air (without vacuum) and with an oxygenator module drawing ambient air by vacuum. With ambient air, SaO_2_ was similar for water (mean ± SD, 27.7 ± 2.1%) and growth medium (mean ± SD, 27.6 ± 4.1%). The oxygen levels ranged from 21.9% to 36.4%, which is within the normoxic range for some tissues^28–31^.

For blood oxygenation profiles, the aim was for physiologic oxygenation conditions (e.g. SaO_2_ = 100%, PaO_2_ = 100^28^). These levels were most closely achieved with low-permeability PharMed tubing without the oxygenator (median ± IQR, 128.0 ± 14.0 mmHg). When using blood in the oxygenator, the closest-to-physiologic oxygenation values were achieved when priming the system with blood (perfusion at 10 mL/min without vacuum back pressure [median, 122 mmHg]). Even with ambient air and no supplemental oxygen, PaO_2_ levels ranging 165-254 mmHg were achieved when vacuum pressure was applied. These supra-physiologic levels can be acceptable in the *ex vivo* setting since liver perfusion systems used to maintain organs during transport between donor and recipient use PaO_2_ > 400 mmHg^32^ and some tissue constructs show enhanced function at higher oxygenation levels^33^. Perfusing blood through the oxygenator yielded statistically higher oxygen levels compared to through silicone tubing alone.

Hemoanalyzer data showed that RBC, HGB, and HCT levels were maintained or increased throughout perfusion. Post-perfusion increases may be due to evaporation or RBCs collecting in higher concentration near the sampling port but the pre- and post-oxygenation values, are practically the same. The RBC, HGB, and HCT levels demonstrate that red blood cells exhibit minimal or no destruction during the oxygenation process as the blood moves through the FABRICA bioreactor, tubing, and oxygenator. Future studies should investigate the status of the blood cells over longer terms to determine how long the blood will remain viable during the course of days to weeks-long perfusion experiments.

### Hemoglobin oxygen saturation can be achieved using the oxygenated FABRICA bioreactor

This study used packed RBC blood, which was more readily available. However, since the i-STAT analyses of packed RBCs do not provide the hemoglobin saturation reading of the blood, a preliminary study of whole blood was performed and determined that hemoglobin is in fact saturated with oxygen during oxygenated perfusion through the system at all flow rates and pressures (data not shown)^28^.

While oxygenation of blood with silicone tubing with and without the oxygenator reached supranormal levels, a strength of this study is the achievement of normal blood PaO_2_ levels with only PharMed low-permeability tubing and ambient air. This simplifies the necessary components of the FABRICA oxygenation system, eliminating the need for supplemental oxygen or an oxygenator module, thereby increasing cost-efficiency. However, since different tissues require different perfusion and oxygenation provisions^30,31^, it is critical to continue modifying the perfusion setup to achieve a greater oxygenation range and control. Since baseline SaO_2_ was not obtained for water or growth media, comparisons to non-perfused blood at baseline could not be made and are out of the scope of this study.

### Practical limitations call for higher operating vacuum pressures

There was no air leak from the FABRICA bioreactor system under any vacuum pressure. However, at low vacuum pressures for blood, air bubbles would slowly accumulate in the oxygenator, causing air pockets in the tubing that would require manual clearance by occluding the intake stopcock valve until air bubbles cleared from the oxygenator. This was labor-intensive and not practical for long-term experiments. This problem was less severe at higher vacuum pressures (-25.40 to -33.86 kPa). Since many blood PaO_2_ values were comparable at higher pressures, using perfusion parameters that do not require manual clearance so that perfusion studies can be performed in the long-term unattended is recommended.

The non-existent or weak correlation between vacuum pressure or flow rate and oxygenation may be due to the oxygen transfer rate from the oxygenator being near or at capacity at these pressures/flow rates.

### The Oxygenated FABRICA exhibits perfusion partially decoupled from oxygenation

Bioreactors should provide predictable, well-controlled, well-distributed perfusion and oxygenation to biofabricated tissues^23^ and it is advantageous if the two could be decoupled from one another so that various perfusion/oxygenation profiles needed to meet specific metabolic demands of different tissues can be achieved. Additionally, for bioreactors to become ubiquitous, particularly in the study of 3D tissues, the bioreactor system should be able to accept and perfuse/oxygenate tissues from different bioprinting platforms. Currently, the oxygenated FABRICA provides controlled perfusion that can be partially decoupled from oxygenation so that oxygenation remains relatively stable while perfusion (volumetric flow rate) is adjusted. This means that bioreactor profiles can be partially tuned to the specific metabolic demands of each tissue type. The complete decoupling of perfusion and oxygenation, which can be achieved with an evacuated chamber surrounding the FABRICA or the incubator, is the goal of future projects but is beyond the scope of the current proof of concept.

### The Oxygenated FABRICA is a simple and robust solution for culturing biofabricated tissues

Since the system is bioprinter agnostic^1^, the oxygenated FABRICA is a suitable platform for culturing tissues generated in nearly any type of bioprinter. The use of house vacuum air instead of a dedicated oxygen tank simplifies the system’s application by reducing the need for complicated or expensive gas equipment. Further simplifying oxygenation is the ability of the FABRICA to be oxygenated with silicone tubing without an additional oxygenator or vacuum back pressure. The provision of media perfusion and oxygenation together enables the FABRICA to provide biofabricated tissues *in vitro* with an *in vivo*-like environment. Given previous successes perfusing bioprinted tissues using the FABRICA bioreactor, future studies will compare the influence of oxygenated perfusion media with known oxygenation levels on the biological outcomes of engineered tissues cultured in the FABRICA bioreactor. Future studies will also attempt to generate low oxygenation levels needed for some tissue types such as bone and investigate if oxygenation stability at these levels is coupled to perfusion flow rate. Additionally, we recently generated self-supporting perfused (SSuPer) tissues^27^, which possess a microchannel structure conducive to perfusion of oxygenated media, and performed computational analysis in order to determine the ability of oxygen to reach the tissue bulk between the channels since the maximum tissue thickness should be less than the maximum perfusion distance of the perfused metabolites, specifically oxygen^34^. Since oxygenation, structure, and perfusion parameters change with cell/tissue type, cell concentration, and cell density, the oxygenated FABRICA coupled with the SSuPer Tissue represent a developing solution for generating robust engineered tissues with *in situ*-like structure which can be cultured *in vitro* with *in vivo*-like conditions.

## Conclusion

Oxygen provision is critical in natural tissue metabolism and should therefore be a feature of bioreactor platforms used to culture biofabricated tissues. This proof of concept study shows that, using ambient air, easily controlled flow rates, controllable vacuum backpressure, and tubing with specific oxygen permeabilities, we can reproducibly and reliably provide a perfused and stably-oxygenated environment within the FABRICA bioreactor. Notably, perfusion and oxygenation can be provided in concert but semi-independently of one another, a critical feature when needing to provide specific culture conditions to specific tissue types. Given that the FABRICA is agnostic of printing method and it possesses the ability to control so many metabolism-critical parameters in one easily implemented system, the modular FABRICA bioreactor platform is developing into a platform with a wide range of direct applications in tissue engineering for a wide range of tissues and will lead to future experiments with more realistic, near-physiologic tissue culture conditions.
